# Application of the NEOH Framework for Self-Evaluation of One Health Elements of a Case-Study on Obesity in European Dogs and Dog-Owners

**DOI:** 10.3389/fvets.2018.00163

**Published:** 2018-07-20

**Authors:** Alberto Muñoz-Prieto, Liza R. Nielsen, Silvia Martinez-Subiela, Jovita Mazeikiene, Pia Lopez-Jornet, Sara Savić, Asta Tvarijonaviciute

**Affiliations:** ^1^Interdisciplinary Laboratory of Clinical Analysis Interlab-UMU, Regional Campus of International Excellence ‘Campus Mare Nostrum’, University of Murcia, Murcia, Spain; ^2^Section for Animal Welfare and Disease Control, Department of Veterinary and Animal Sciences, Faculty of Health and Medical Sciences, University of Copenhagen, Copenhagen, Denmark; ^3^InMedica Vilnius–Alfa Clinic, Vilnius, Lithuania; ^4^Department of Oral Medicine, Faculty of Medicine, University of Murcia, Murcia, Spain; ^5^Department for Serology, Immunology and Biochemistry, Scientific Veterinary Institute “NoviSad”, Novi Sad, Serbia

**Keywords:** obesity, one health, evaluation, canine, dog, human

## Abstract

Obesity is a malnutrition disorder of global concern with increasing prevalence driven by underlying societal, economic and environmental mechanisms leading to changed physical activity patterns, eating behaviors and diet compositions in both humans and in their pet-dogs. A questionnaire-based study was carried out as a joint effort across 11 European countries. It was considered a One Health (OH) initiative between scientists from human and animal health sectors aiming to identify factors associated with obesity in dog owners and their dogs. Expected outcomes of this approach included new insights unachievable by single-sector research initiatives, and hence potentially leading to new cross-sectorial solutions. We performed an internal evaluation among the actors of the obesity initiative using the framework for evaluation developed by the “Network for Evaluation of One Health” (NEOH). It served as a case-study for the NEOH consortium to illustrate the application and provide feedback on the utility of the framework. The evaluation was performed by a subgroup of scientists also involved in the obesity study group, and it consisted of: (1) the definition of the initiative and its context, (2) the description of the theory of change, and (3) the qualitative and quantitative process evaluation of operations and supporting infrastructures scored on a scale from 0 to 1. In the One Health operations, the obesity study initiative scored medium high on OH-thinking (0.5) and OH-planning (0.45), and relatively high on OH-working (0.7). The supporting infrastructure score was high for systemic organization (0.8), but low for sharing (0.45) and learning (0.28). The calculated OH-index was 0.29 (on scale 0 to 1) indicating that the full potential of health integration and collaboration was not exploited in the initiative, and the main issue identified was a lack of stakeholder engagement. The OH-ratio of 1.1 indicated equal focus on operations and supporting infrastructures. Hence, the evaluation identified potentially counterproductive as well as beneficial characteristics, which are further discussed in this paper in relation to the expected outcomes. The NEOH framework for evaluation requires that the evaluators have a good understanding of systems thinking and the mechanisms of the health issue targeted by the initiative.

## Introduction

Today, obesity is considered the most frequent malnutrition disorders in many parts of the world. It is increasingly recognized as a “wicked problem,” because it is highly complex and resistant to resolution with no clear stopping points. Furthermore, attempts to solve it might reveal or create new problems, because it is a symptom of other underlying problems with systemicsocietal, environmental and economic drivers (1). Obesity is associated with different pathologies including orthopedic and respiratory diseases, endocrinologic and oncologic disorders, compromised well-being, and decreased life-span (2, 3). In addition to the increasing societal burden of obesity related to the increasing health expenses, disabilities, reduced life-expectancy and productivity losses, there appears to be detrimental environmental impacts such as increasing emissions of greenhouse gases associated with increasing population rates of obesity (4). All of this is a growing concern, since the prevalence of obesity is continuously increasing in both dogs and humans (5, 6). Hence, all potential obesity mitigation opportunities should be explored, including links between and factors explaining the links between obesity in dog-owners and their pet-dogs. Studies have shown that such links are relevant with common underlying environmental factors including physical activity patterns, eating behavior and diet compositions likely to be driving the development in both species (7, 8). Even though initiatives have been carried out and are on-going to mitigate the obesity development in humans, and in pets, respectively, transdisciplinary approaches bridging different disciplines and health sectors, e.g., human and veterinary medicine, sociology and psychology and targeting both species simultaneously through joint interventions are likely to be more effective or at least to contribute to improved mitigation of the obesity trends (9). Such transdisciplinary efforts including focus on human behavioral changes benefitting animals as well as the environment are often referred to as One Health (OH) initiatives, when they are aiming to achieve improved human and animal health and welfare simultaneously. Kushner et al. (9) demonstrated the benefits of a combined people and pet weight loss program. However, dog-ownership has hitherto mainly been investigated as a tool for human health status improvement. An example of this is the study by Wohlfarth et al. (10) that illustrated significantly higher level of some types of physical activity in obese children between 8 and 12 years old, who were enrolled in a comparative intervention trial with dogs vs. human co-performers of movement tasks.

Aiming to identify social, environmental and economic drivers of obesity in dog-owners and their dogs, a questionnaire-based study was performed to collect and analyze information on self-reported body mass parameters, physical activity, eating patterns, diets and diseases in both humans and dogs, as well as perceptions of the dog owners by scientists related to both human and pet health sectors in 11 European countries. Potential cross-sectorial solutions with added value toward obesity mitigation were the main targets of that study, which will be referred to in this paper as “DODOS” (i.e., *D*og *O*wner And *D*og *O*besity *S*tudy). Because of the joint research team being multidisciplinary (Table 1) and because the target was the detection of social and environmental drivers of obesity in two populations, dogs and their owners, this research initiative could be considered an example of a OH approach to a health challenge that not only occurs in at least two species, but also seem to be linked by common factors related to the two species. We therefore used DODOS as a case study of an OH initiative about a non-communicable disease for illustration and evaluation of a new framework and tools developed to facilitate evaluation of OH initiatives. Over the last decade, there has been growing interest for the OH approach implementation in the health research, systems and services, since mutual benefits are expected in comparison to single-sector approaches—also referred to as “silo-approaches” to health issues (11). The benefits include improvement in animal-, human-, and eco-health and well-being, higher quality or larger quantity of relevant information and economic efficiency (11). However, no validated science-based evaluation protocols for quantitative measurement and evaluation of OH activities have previously been available. In order to fill this gap, a “European Union Action on Coorporation in Science & Technology” (EUCOST Action, TD1404) “Network for Evaluation of One Health” (NEOH) designed science-based guidelines for qualitative and quantitative evaluation of OH-initiatives. The NEOH framework (12) is recommended for external evaluation of OH-initiatives. However, it might also provide useful information and feedback, if used for self-evaluation within an initiative.

**Table 1:** Participating countries and involved specialists in the DODOS study evaluated in this manuscript with indication of the represented disciplines and sectors.

**Nr**.	**Country**	**Involved Specialists**
1	Croatia	Clinical pathology specialists (VM)
2	Denmark	Epidemiologist (VM) Endocrinologist (VM)
3	Italy	Clinical pathology specialists (VM) Internal medicine specialist (VM)
4	Lithuania	Anatomy specialist (VM) Physiology specialist (VM) Pulmonologist–pediatrician (HM)
5	Poland	Reproduction specialist (VM)
6	Portugal	Anaesthesiologist (VM) Biologist PhD student (VM)
7	Rumania	Reproduction specialist (VM)
8	Serbia	Immunology specialist (VM)
9	Spain	Clinical pathology specialists (VM) PhD student (VM) Odontologist (HM)
10	Sweden	PhD students (VM)
11	Turkey	Cardiologists (VM)

*VM, veterinary medicine; HM, human medicine*.

The aim of the present work was to perform an internal evaluation (i.e., a self-evaluation) of the DODOS initiative using the NEOH evaluation framework and tools to improve the learning about essential operations and infrastructures of OH-initiatives within the DODOS consortium. It also served as a case-study for the NEOH consortium to illustrate the application of the framework to a non-communicable disease, and to gain feedback on the utility of the framework and tools for further improvements.

## Material and methods

In the DODOS, a questionnaire about the perceptions of dog-owners about human and dog obesity, factors associated with and potential drivers of obesity was designed and distributed to dog-owners in 11 European countries by means of personal contacts, veterinary clinics and social media during the period December 2016 to March 2017. The questionnaire contained 74 questions, and apart from demographic questions and questions about health status in the dogs and their owners, many questions were on a Likert scale to assess perceptions of different statements about eating behavior, diets, physical activity and perceptions about the dogs and the dog-human relationship that could be related to obesity development. In total, 3185 questionnaire responses from 10 of the 11 study countries with a sufficiently high number of valid responses were included in multivariable statistical analyses. Relevant perceptions, physical, socioeconomic and environmental factors associated with obesity in dog-owners and their pet-dogs were identified. The OH evaluation of DODOS was initiated a few months after the initiation of the DODOS. However, at the time of the performance of the last part of the evaluation, the data collection and statistical analyses were finalized, and the DODOS was reported in a manuscript submitted for peer-review in an international scientific journal. However, the results of the DODOS will not be covered in this manuscript except where directly relevant for the evaluation, as they are not the main focus of the OH evaluation.

The NEOH framework includes four overarching elements (12). However, only elements one to three were carried out for DODOS, i.e., (1) the definition of the OH initiative and its context (i.e., the system, its boundaries, and the OH initiative as a subsystem); (2) the assessment of expected outcomes based on the theory of change (TOC) behind the initiative and (if possible) unexpected outcomes emerging in the context of the initiative; and (3) the process assessment of the operations and supporting infrastructures, also known as the “OH-ness” of the initiative. Element (4), assessment of the association between the degree of “OH-ness” and the outcomes produced, could not be performed because it makes most sense to evaluate element (4) by comparing across several case studies, a task that the NEOH consortium will work on after the framework has been used for many case studies.

The background theory and each element of the NEOH framework are described in detail by Rüegg et al. (12) and a supplementary Microsoft Excel file is provided online and can be used as a template for the evaluator(s) to fill in when going through the process evaluation in element three. The system leading to human and pet-dog obesity was described by the first and last authors of this manuscript by building partly on their experience with pet-dog obesity from veterinary clinical practice and research, and partly on literature search on the system boundaries and linkages relevant for the development of obesity in humans. The TOC was deducted by logical reasoning combined with literature suggesting or illustrating the benefit of a joint effort between human and pet-dog scientists in the obesity context.

Element three was an internal evaluation mainly performed by the first two and the last authors of this manuscript, even though the interpretation of the points to be evaluated in the tool was discussed with the other authors and NEOH consortium members during the process. To be able to fill in the provided NEOH tool for the “OH-ness” evaluation and to assess the outcomes of the DODOS, the core scientific members, i.e., the 24 authors of the manuscript reporting on the DODOS, were in late May-early June 2018 asked to respond to an anonymous online questionnaire containing 20 questions about thinking, planning, working, sharing, learning and systemic organization as well as expected and unexpected outcomes. The questionnaire with introduction text, questions and frequency distributions of answers as well as written answers to open ended questions are available in Supplementary materials 1. The means of communication were otherwise mainly through email and on-line meetings with individual scientists from the different participating countries during the case study period.

## Results

This section provides an overview of what was found and deducted about the three first elements in the NEOH framework, in other words what the evaluators found relevant and true for the obesity case study based on the input provided by DAODOS actors and the experiences gained during the obesity case study period. It does not contain results from the obesity study itself, except where it is considered relevant to understand how the results were obtained or deducted in the evaluation.

### System definition and the initiative within the defined context

Obesity is a global epidemic health problem, for which all the dimensions of the system are highly interconnected. The following dimensions were considered in the context: *Geographical space* a highly important dimension since obesity drivers are present worldwide even though they can vary between countries. The dimension of life is an important dimension with obesity drivers acting at all scales (i.e., at cell, organ, individual, population, regional, national society, international level). *Network/organization* is a highly relevant dimension as obesity drivers and potential solutions exist at the individual, institutional, national and international levels. *Economic* drivers are important for the development of obesity. In particular socioeconomic status is important to consider, but other economic drivers of obesity can also be identified including economic incentives for companies producing obesinogenic products, transportation means etc. *Time* is an important element of obesity development both for the individual (e.g., child obesity vs. developing obesity over time due to too prolonged high caloric intake and lack of physical activity) and at societal level. The changes in drivers over time are also important to consider. *Governance* is relevant as it can provide means to prevent obesity development, e.g., by dictating development toward less obesinogenic environments in society and by allocation of funds to prevent obesity and reduce consequences of obesity through research, innovation and intervention. It might also be used to impact the economic driver through e.g., sugar or fat taxes. However, governance of health issues and potential solutions related to the global obesity epidemic are currently highly segregated into separate sectors rather than cross-sectorial and today very little obesity prevention and mitigation is based on transdisciplinary research and development (13).

**Figure 1:**
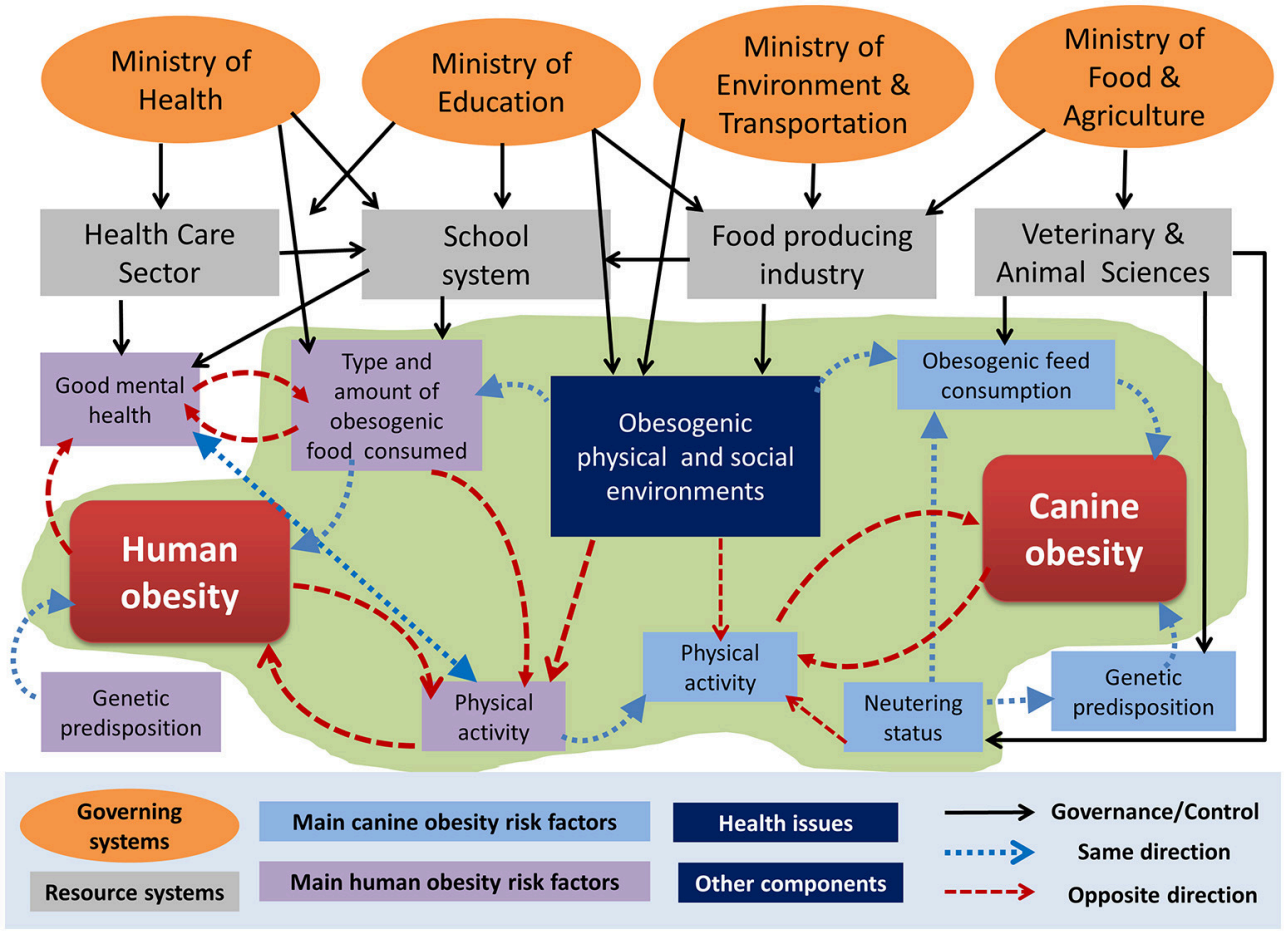
Visual representation of the context of human and pet-dog obesity including linkages and feedback loops in the system. The list of ministries and resource units is not exhaustive and the names are examples as these vary between countries as well as over time within countries. A green shaded area covers the elements considered in the initiative under evaluation.

A conceptual illustration of how the obesity development in dogs and dog-owners can be perceived as interlinked is provided in Figure 1. The context description was inspired by, but does not cover the full complexity of obesity described in, the UK Forsight Governmental project systems maps of obesity published online in 2007: https://www.gov.uk/government/publications/reducing-obesity-obesity-system-map, accessed 12 June 2018). The UK Forsight systems map illustrates individual, socioeconomic and environmental drivers, elements and feedback loops affecting obesity in humans. However, for the DODOS the animal component and animal-human bond was important and not considered in the UK Forsight systems map. The food and animal feed industry as well as food and feed consumption patterns have strong potentials to negatively affect the health of both humans and pets, even though they are usually governed (if governed at all) through different ministries in traditional sectorial governance structures. Likewise, psychological factors and obesogenic environments that affect physical activity and eating behaviors in both humans and their pets are generally only considered in the human health care system, even though there might be a potential for prevention of obesity development by utilizing the interlinked driving factor between the sectors. Two obesity drivers, social and economic, are frequently described in literature. For instance, the obesity is related with the sedentary way of living and ingestion of hypercaloric food, among others (14). Also, low incomes have been associated with malnutrition resulting in obesity; and the obesity and obesity-related diseases result in increased expenses (15).

The initiative is, however, mainly targeted at social drivers, i.e., perceptions and behaviors that can affect the social aspects of obesity, since the main stakeholders involved in the study were dog-owners, researchers, health professionals and clinicians. The following actors and stakeholders within the system illustrated in Figure 1 were included in the obesity case study: investigators (human and veterinary medicine, health science researchers); clinicians (human and veterinary health specialists) and a biologist as well as dog-owners.

### Theory of change (Toc) and expected and unexpected outcomes/impacts

**Figure 2:**
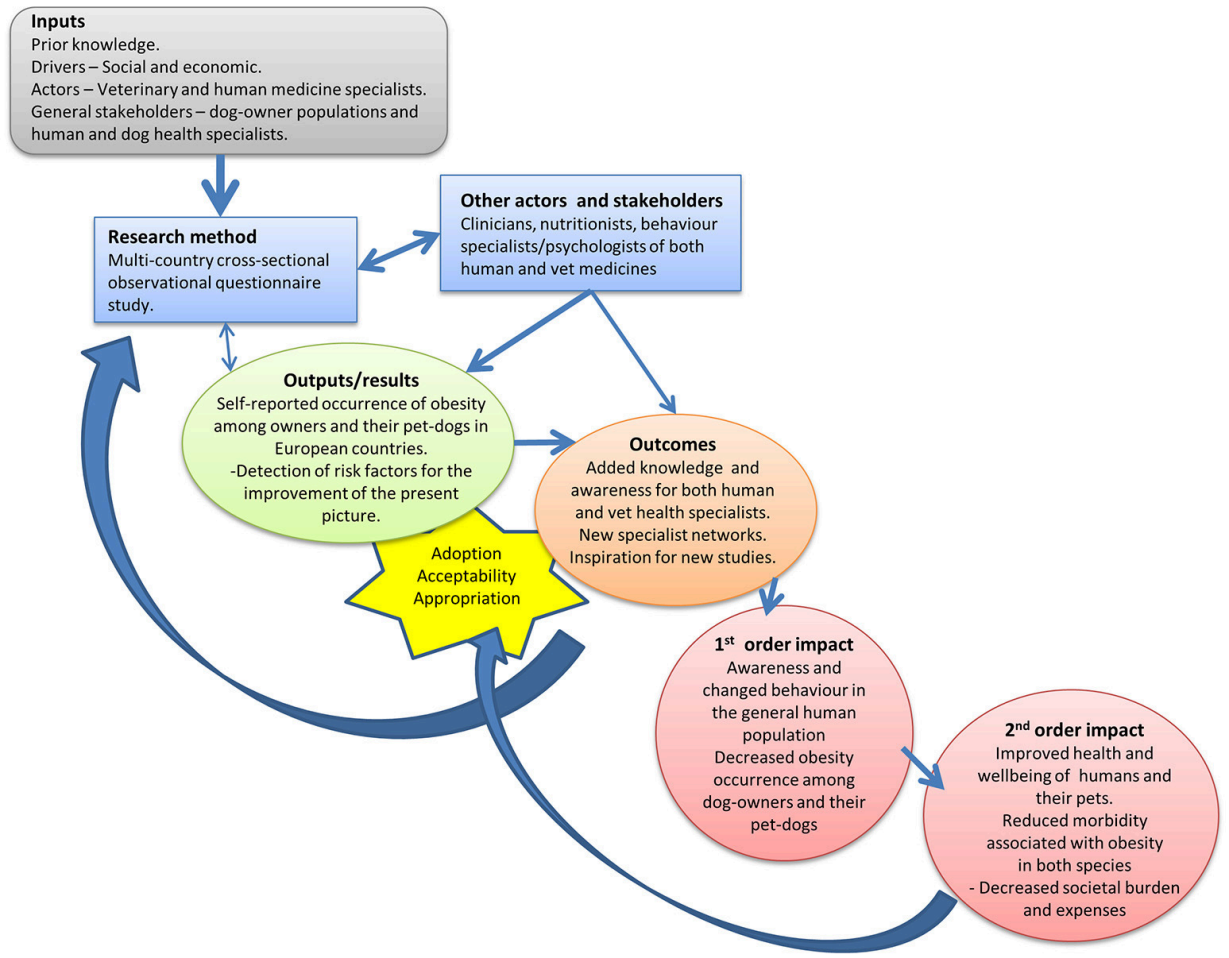
The schematic presentation of the theory of change of the dog-owner and dog obesity study (DODOS).

The primary long-term goal of the DODOS initiative was to contribute to decreasing obesity occurrence among dog-owners and their pet-dogs. This would lead to a second order long-term impact of improved health and well-being and reduced morbidity associated with the obesity in both species, finally resulting in decreased societal burdens and expenses. In order to reach these impacts, required inputs such as prior knowledge, human resources for research, research methods and materials, actors and stakeholders from multiple disciplines and sectors (Table 1), outputs such as questionnaire and analysis results, and outcomes such as improved knowledge, new collaborative networks and new solutions being created based on these (Figure 2).

The output will mainly be the communication of the results in publications and presentations at conferences to the scientific community, and to the public through layman communications, which would then lead to the expected outcomes (increased knowledge in the relevant populations). Some outcomes were anticipated directly as a result of DODOS, but also unexpected outcomes were mentioned by the actors in the evaluation questionnaire (Table 2). The first and second order impacts will depend on the uptake of the outcomes including changed governance procedures, changed behaviors in dog-owners which may affect their dogs and other humans as well, with consequential health improvements and long-term effects thereof. The fact that many households have pet-dogs provides a strong basis for creating change, if the new knowledge from the DODOS and other studies to follow is utilized.

**Table 2:** Dog-owner dog obesity case study (DODOS) outputs, expected and unexpected outcomes and impacts according to direct communication and responses to an online questionnaire for actors in the DODOS consortium.

Disciplinary outcomes and outputs	- New collaboration partners - Knowledge about risk factors for obesity in humans and dogs, respectively - Inclusion of the study results in a PhD thesis
Inter-disciplinary outcomes and outputs	- A scientific paper or report being published - New collaboration partners across disciplines - Identification of risk factors for obesity that bridge two species - Knowledge about perceptions in dog-owners that may affect the development of obesity in both humans and pet-dogs
OH outcomes and outputs	- Comparison of factors affecting obesity in dog-owners and in dogs leading to a better understanding of underlying factors that might not be directly measureable - New linkages between collaboration partners across disciplines and sectors in different countries of Europe - An improved interest of participants in OH approaches - Experience with international collaboration and team-work - Learning about the planning and organization of future One Health initiatives - Experience useful to improve the study design for future obesity studies at the human-animal-environment interface - Awareness of direct and indirect obesity drivers and consequences, both among animals and owners - Ideas and plans for new projects
Un-expected outcomes and outputs	Actors highlighted unexpected outcomes for the following points: - perceptions among dog-owners, e.g. that obesity is not considered a disease by all people and that not all consider the OH approach plausible to combat obesity - Actors learning about opportunities as well as biases and other study design challenges in questionnaire studies involving social media for recruitment of respondents - Actors learning about complicated publication processes

### Assessment of OH-NESS of the initiative

**Figure 3:**
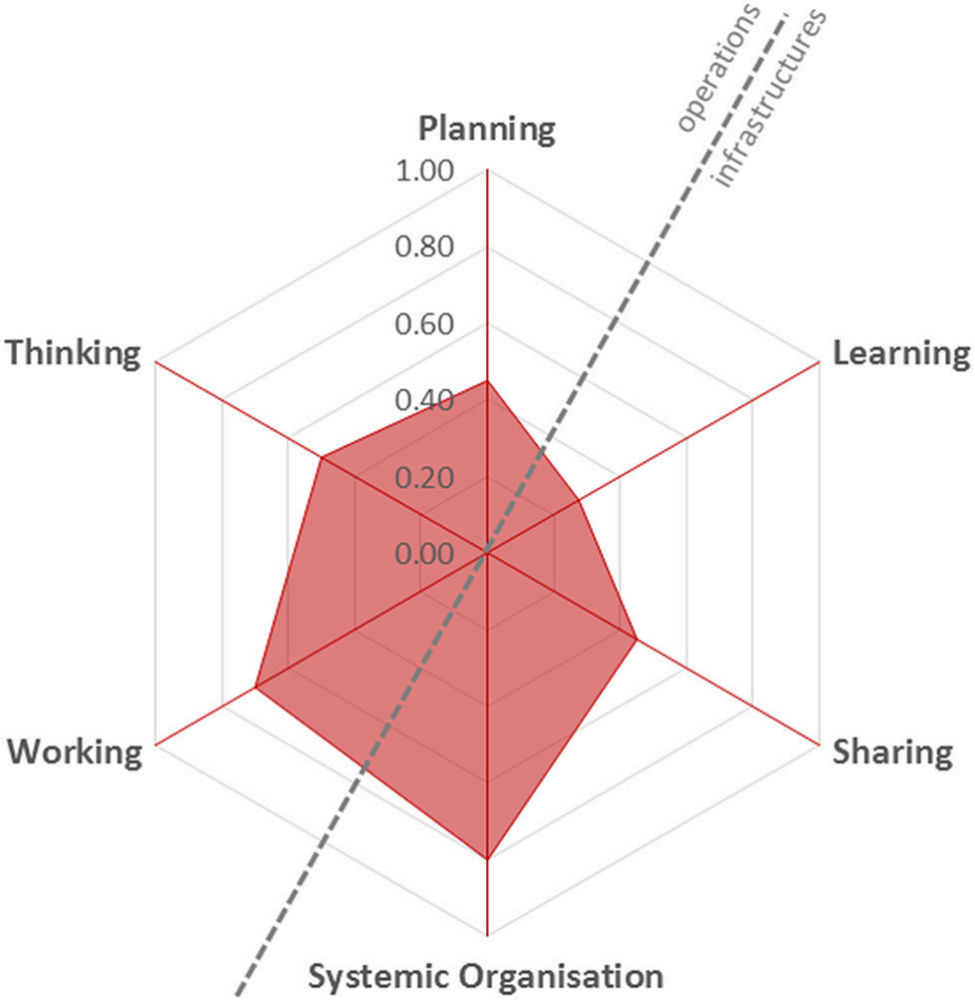
The NEOH evaluation spider diagram indicating overall scores for six One Health characteristics of a case study on obesity among dogs and their owners in 11 European countries.

The results of the qualitative as well as quantitative assessments for each point in the NEOH evaluation framework and tool can be seen in the supplementary Excel-file for the obesity case study. The evaluation points are fixed by the framework, but we have supplied comments relevant for each evaluation point in six spreadsheets about each of the characteristics of OH initiatives to be assessed, i.e., the main OH-*operations*: thinking, planning, working, as well as the *supporting infrastructures* (learning, sharing and system organization). In brief, the OH-thinking in the evaluated initiative was reflected by the multiple dimensions mentioned above, because it evaluated the obesity problem in both humans and dogs and in two life dimensions—individuals and populations; and two geographical dimensions—individual country and Europe. The DODOS scored 0.5 on OH-thinking. The OH-planning of this study was led by one person supported by a specialists composing core committee, who contact the rest of the responsible persons in each country. OH-planning initiative scored 0.45. OH-working, scored 0.7, was reflected by the multidisciplinary collaboration (human medicine, veterinary medicine and a biologist) and inclusion of stakeholders (owners and clinicians) to the problem evaluation and possible ways of its solving. For OH-learning the score was low at 0.28 indicating limited adaptive and generative learning within and outside the initiative, and for information and data sharing the score was 0.45. Systemic organization which is mainly indicative of team-work organization and leadership was scored high at 0.8. Figure 2 illustrates the OH-scores and OH-index, which was 0.29 (on a scale from 0 to 1) indicating that the full health integration and collaboration potential suggested by NEOH for OH-initiatives tackling complex problems was not exploited in the DODOS study. The ratio between operations and supporting infrastructures was 1.1 indicating a balanced focus on operations and infrastructures (Figure 3).

## Discussion

To date only small-scale studies have indicated associations between obesity in dog-owners and obesity in their dogs and assessed potential causal factors (8, 9, 10). With this in mind, a large scale, multinational study was designed aiming to identify dog-owner perceptions, potential causal factors and behaviors of the owner relative to his/her pet that might lead to or increase the risk of canine obesity as well as his/her own obesity. As highlighted above, the human-animal bonds are usually overlooked in existing obesity context description (7, 16, 17). The main aim of OH-strategies is to improve health and well-being across different species and their environment (or ecosystem) through targeted collaboration between disciplines and sectors, and the involvement of essential stakeholders is important in OH-initiatives. This often includes engagement of relevant groups of citizens. Although in recent years OH-based studies have increasingly gained attention in scientific literature, no validated guidelines for quantitative measurement of OH-activities have been available previously, and prior obesity initiatives have not been evaluated with focus on OH-approaches. Hence, the NEOH evaluation framework provided an interesting opportunity to learn about shortcomings and beneficial aspects of the DODOS initiative. Both objectives of the present evaluation study were achieved, namely to evaluate the DODOS study and consortium as well as to assess the usefulness of the framework for OH-evaluation.

Psychological factors and obesogenic environments affect physical activity and eating behaviors (18), but human-animal bond related physical activities are frequently not considered in the human health care system even though there are potentially strong obesity preventing measures that would be easy to apply in other sectors, e.g., dog-play activities for children as well as dog-assisted physical activities for adults, “health schools” in which humans can learn about healthy food consumption practices through the learning about appropriate diets for healthy dog and humans. Hence, some OH-initiatives for reducing obesity in both dogs and dog-owners clearly build on learning as an important element in the OH-initiative. Unfortunately, it became evident that OH-learning was the element in the DODOS study that was scored lowest mainly due to lack of learning infrastructures among stakeholders and actors which would go beyond basic learning and support adaptive learning, i.e., learning that focuses on correcting or improving existing procedures, processes, competences and technologies, as well as generative learning, i.e., learning that focuses on questioning the existing norms and that encourages to see beyond the existing situation to generate new paradigms. One way this could have been improved in DODOS would have been to engage stakeholders as well as decision makers in governing institutions early on, i.e., during the planning process as well as during the dissemination process during and after the study period.

The moderate OH-thinking score suggests that the lack of stakeholder engagement might have been grounded in a lack of general acknowledgment in the DODOS consortium that addressing more of the obesity context including feedback loops that could have been targeted in the initiative might have created other outcomes and led to a larger impact of the initiative. During the DODOS design and planning the OH-thinking was less accepted by human health specialists, and the majority of the approached specialists did not consider the obesity to be a health problem for pets and seemed to think that transdisciplinary solutions were not efficient or not possible to perform. For this reason, the majority of actors driving the DODOS were veterinary specialist with scientific and/or practical experiences. This was one of the main limitations for implementing an OH approach.

Carrying out an OH-evaluation using the NEOH framework requires a good understanding of systems thinking and OH in general. Stakeholder involvement in the evaluation is required, since a lot of information related to study objectives, planning of the work and the way of working such as data analysis, sharing of information and leadership is needed. This particular evaluation was performed by several of the DODOS actors as a self-evaluation, which might have introduced some biases. Clearly there were differences in how well the processes, outputs and outcomes of the initiative were known and understood by the 21 actors who responded to the actor questionnaire. This would probably have been less of an issue, if more/better sharing and learning infrastructures had been ensured in the initiative, but might also be related to economic and human resources and leadership choices made during the study design, performance and finalization.

The application of the evaluation questions and tools provided by NEOH allowed identifying strengths and limitations of the case study with regards to the OH approach. These strengths and limitations might impact the ability of the study to achieve some added value compared to disciplinary projects within the topic obesity in dogs and humans. An OH-index of 0.29 out of a possible total of 1 indicates that several indicators did not achieve high scores. However, it is difficult to say whether this has an impact on the desired outcomes of the study. This remains to be investigated when the OH-index is compared across different initiatives in the future. An OH-ratio of 1.1 indicated that infra-structures underpinned the operations in the initiative even though this value also has to be seen in relation to the scores of each of the elements learning, sharing and systemic organization which were not all scored high for DODOS. Limitations were mainly noticed in the thinking, sharing and learning parts of the evaluation, and in the identified outcomes.

Information sharing is described to be one of the basic criteria for OH-studies (19). Although data sharing occurred in DODOS, it was uni-directional and the full raw data set was only available for the core committee. However, later the results summary reported by core committee would be discussed and was planned to be analyzed by all the participants in order to achieve the holistic approach of the obesity as a disease, in this way aiming to improve the sharing in DODOS. One option would be to disseminate the new knowledge and information from the study through the same channels that were used to recruit dog-owners to reply to the DODOS questionnaire.

One of the fundamentals of OH-studies is to obtain higher impact of outcomes in comparison to conventional single sector or single discipline approaches in terms of improved health and well-being, and reduced economic costs or improved cost-benefit ratios (20, 21). This could not be directly measured from this study. However, other outcomes were identified by the actors in the actor questionnaire (Supplementary materials 1, Table 2), mainly related to the following overall categories: scientific knowledge and understanding of factors associated with obesity in dogs and dog-owners, increased awareness of the linked obesity issue between pets and pet-owners, learning about study design and publication processes in large international consortia which is an important capacity building aspect, improved knowledge and understanding of OH approaches and important new collaboration linkages across the consortium. However, stakeholder involvement was not deemed sufficient for the initiative to have a certain societal impact at the point of evaluation. This is an important point for the consortium members to consider, not only for the DODOS, but also in future research initiatives within the field. However, as emphasized by Bartges et al. (17) this requires “efforts and leadership of a committed group of like-minded individuals representing a range of scientific and medical disciplines. Interested parties will need the means and opportunities to communicate and to collaborate, including having the resources and funding for research.” In fact, resources for engagement of actors and stakeholders were very limited in the DODOS and were mainly build on voluntary engagement.

In conclusion, the utility of the evaluation tools for the evaluation and potential improvement of OH-initiatives was illustrated. Short-comings in critical elements were identified in DODOS. It would have been useful to use the NEOH evaluation framework and the evaluation tools as a checklist during the project design and planning phase since this could help to identify the limitations of the study and consortium composition, which might be corrected before the study begins or during the study period. Moreover, using the framework facilitated targeted communication between all actors in the initiative to gain an improved common understanding of the OH-characteristics. In this particular case study, it might have improved the participation of human health professionals and researcher or stakeholders from other relevant disciplines to have more elaborated discussions about the system description and the TOC before the study was initiated.

## Author contributions

AM-P and AT data compilation and tool complementation. AM-P, LN, SM-S, JM, PL-J, SS, and AT paper drafting and paper and tool revising.

### Conflict of interest statement

The authors declare that the research was conducted in the absence of any commercial or financial relationships that could be construed as a potential conflict of interest.
