# Supplementary material for: Application of the NEOH Framework for Self-Evaluation of One Health Elements of a Case-Study on Obesity in European Dogs and Dog-Owners
Source: Front Vet Sci. 2018 Jul 20;5:163. doi: 10.3389/fvets.2018.00163 (PMC6064947; doi:10.3389/fvets.2018.00163)
Supplement: Supplementary file 1 [file Data_Sheet_1.PDF]

Supplementary material 1: Questions to actors in the One Health initiative: 'A study on European dog-owner perceptions of obesity and factors associated with human and canine obesity'

This questionnaire was developed to collect information for a One Health evaluation of an international research initiative on dog and dog-owner obesity. The questionnaire was sent to the 24 authors of the manuscript entitled 'European dog-owner perceptions of obesity and factors associated with human and canine obesity'.
The respodents were informed that the answers would be used in a summarised form in the about the evaluation of the initiative, with no possibility to track the individual respondents, in a case study illustrating the use of 'the NEOH framework for evaluation of One Health intitiatives' (http://neoh.onehealthglobal.net/)

There are 20 questions and the respondents were given the option to add their own answer options, if they could not use the predefined options.
It was estimated to take 20-30 minutes to answer the full questionnaire.

1) Think about how you perceive the complexity (the context) of and the background behind the development (the drivers) of obesity in dogs and dog-owners. How well do you think the obesity study reflected the system complexity of the obesity issue in dogs and dog-owners?

It covered ALL essential elements behind obesity in dogs and dog-owners	5
It covered MANY essential elements behind obesity in dogs and dog-owners	12
It covered SOME essential elements behind obesity in dogs and dog-owners	4
It covered FEW essential elements behind obesity in dogs and dog-owners	0
It covered NO essential elements behind obesity in dogs and dog-owners	0
Other	0

2) Were different stakeholder perspectives pursued and used in the development of the study hypotheses and study design?

Yes, to a large degree. Stakeholders were consulted a lot about the design of the study.	3
Yes, to some degree. Stakeholders were consulted about the design of the study.	8
Perspectives were considered, but I do not think stakeholders were consulted about the design of the study.	3
No, as far as I know stakeholder perspectives were not pursued and stakeholders not consulted.	1
I do not know.	5
(Blank)	1

3) Was there a planning process leading to an appropriate number of actors/authors with appropriate discipline and experience backgrounds in the group of people performing the obesity study?

Yes	9
To some extent	7
No	1
I do not know	4
Other	0

4) Did stakeholder engagement ensure a One Health approach during the working and organisation of the obesity study initiative?

I consider it a One Health approach with stakeholders of human, animal and environmental health engaged	7
I consider it a One Medicine approach with no environmental health stakeholders engaged	5
I consider it a partial One Health approach with human, animal and some environmental health stakeholders	5
I consider it an interdisciplinary approach without stakeholder engagement	0
I do not know	4
Other	0

(Supplementary materials 1 continued)	
**5) Did lack of funding for research time for many of the involved actors impact the final study outcome or quality?**	
Yes	1
To some extent	4
No	9
I do not know	5
Other: Yes, but much was achieved by voluntary efforts (spare time efforts of researchers).	1
Other: It is posible but I do not known how others handled the time issue.	1
**6) Did the planning of this study allow for sufficient self-assessment with consecutive improvements of the study design, analyses, performance/results etc.?**	
Yes	15
No	3
I do not know	2
Other: I was not involved in the planning and do not know	1
**7) Did the planning of tasks and resources to fulfill the planned tasks match the objective of the obesity study?**	
Yes	14
To some degree	6
No	0
I do not know	1
Other	0
**8) Do you think a One Health approach is needed to solve the obesity challenge in dog-owners and their dogs?**	
Yes	17
To some degree	4
No	0
I don't know	0
Other	0
**9) Do you think the non-scientific community (citizens) have been sufficiently involved in the obesity study for it to have the expected impact on obesity?**	
Yes	13
No	5
I do not know	2
Other: at this date no	1

(Supplementary materials 1 continued)	
**10) Were all the right disciplines represented in the obesity study? Please write your response in a few words.**	
All disciplines relating with the obesity study were included as much as possible; In my opinion the right disciplines were represented in the study; Yes they were; Yes, I think so; Yes; Yes, the obesity study was performed around medical; veterinary and social approach, all of these are essential to prevent and manage the obesity; I would say yes, a lot different disciplines have helped gather the data for the study.	7
Human medicine specialists were low-represented; Human medicine was underepresented; It was primarily veterinarians; I am not aware that human health researchers were involved; Yes, but some were not well considered.	4
Measurement of anthropometrical parameters and also parameters of obesity in dogs; No, sociologists, socioeconomists, public health and behavioural specialist lacking; Maybe; I would dare say that psychiatry/psychology could also been involved;Biochemical analyses need to be done; Yes but questions formulated in a way that could allow more information about the physical activity habits, particularly of owners and food habits would be useful.	5
A wider selection of dogs breeds should be included [as well as crossbreeds]	1
I do not think this is relevant, since obesity can affect anyone.	1
I do not know as I only know a few co-authors	1
(Blank)	2
**11) To what extent did the different disciplines work together in the obesity study?**	
A lot of of cross-disciplinary collaboration	7
Some cross-disciplinary collaboration	11
Little cross-disciplinary collaboration	1
No cross-disciplinary collaboration	1
I do not know	1
Other	0
**12) To what extent did the different nationalities work together in the obesity study?**	
A lot of of international collaboration	13
Some international collaboration	7
Little international collaboration	0
No international collaboration	0
I don't know	0
Other: I can only answer for myself, and if so, little	1
**13) Was there sufficient sharing of data and information on methods WITHIN the obesity study group?**	
Yes, full or almost full sharing of data and information on methods	11
Yes, some sharing of data and information on methods	8
No, too little sharing of data and information on methods	1
No, there was no sharing of data and information on methods	0
Other: The handling of the written data among countries could have been better stratified and organized on	1
I don't know	0
**14) Was there sufficient sharing of information and results OUT OF the obesity study initiative for it to have a long-term**	
Yes, sufficient sharing with stakeholders	6
Some sharing with stakeholders	5
Little sharing with stakeholders	4
No sharing with stakeholders	2
I do not know	3
Other: So far no sharing with stakeholders in our country	1

(Supplementary materials 1 continued)	
**15) Did the obesity study provide relevant new knowledge in relation to the state of the art and the obesity challenge in dogs and dog-owners?**	
Yes, highly relevant new knowledge provided	14
Some relevant new knowledge provided	6
Little relevant new knowledge provided	1
No relevant new knowledge provided	0
I don't know	0
Other	0
**16) How would you characterise the learning achieved during or as as result of this obesity study?**	
**16 a) Learning at the individual level (your own learning)**	
Basic learning - facts that may not be used/applied later	1
Adaptive learning - will be used to change procedures in the future	12
Generative learning - that change underlying beliefs / norms	6
No learning	0
Don't know	2
**16 b) Learning at the team/group level (authors and close colleagues)**	
Basic learning - facts that may not be used/applied later	2
Adaptive learning - will be used to change procedures in the future	11
Generative learning - that change underlying beliefs / norms	7
No learning	0
Don't know	1
**16 c) Learning at the institutional level (other colleagues/institution)**	
Basic learning - facts that may not be used/applied later	4
Adaptive learning - will be used to change procedures in the future	7
Generative learning - that change underlying beliefs / norms	6
No learning	1
Don't know	3
**16 d) Learning of stakeholders**	
Basic learning - facts that may not be used/applied later	1
Adaptive learning - will be used to change procedures in the future	5
Generative learning - that change underlying beliefs / norms	6
No learning	1
Don't know	8
**16 e) Learning in general society**	
Basic learning - facts that may not be used/applied later	3
Adaptive learning - will be used to change procedures in the future	3
Generative learning - that change underlying beliefs / norms	7
No learning	0
Don't know	8

(Supplementary materials 1 continued)	
**17) What are the most important EXPECTED OUTCOMES of the obesity study of relevans for One Health? Please describe in your own words. The outcomes are not limited to the outputs such as the results of the study and the upcoming publication, but can also include outcomes for you as an actor (e.g. new collaboration partners, new knowledge on which you will build new studies, etc.)** ***(quotes from respondents collated and counted by topic to ease interpretation)***	
4xNew collaboration partners; Certainly it enhanced future international collaborations with peers; Further collaboration with colleagues from Europe; New collaboration partners in my own country;2xNew collaborations possibilities;	8
Possibility to improve study design of research; Data available that might inform future studies with better study designs so that they might have a bigger impact; New information that may be further exlored to get more detailed information about this problem; New studies; New ideas for future project;Possibility to improve study design of further research and collaboration with colleagues from Europe;Plans and new ideas for the future;	7
Interest in One health concept research;Learning about One Health, One Medicine and collaboration challenges in large, international consortia where people have different expectations and very different quality expectations to research studies; Working in an international collaboration was challenging and taught all participants to navigate in a muticultural setting; Working with new colleagues in one team;	4
Two publications; Paper; Part of PhD thesis; Congress presentation;	3
5x'New knowledge'; Risk factors identified; New information that may be relevant for stakeholders; The importance of considering common actions to reduce obesity in dogs and cats; Also this study show differences between countries were the causality for these differences can be investigated in future studies and used in developing possible new strategies for obesity prevention; Results of the study in different countries; Obesity is one of the most important health problems in dogs owner and dogs;Owner life style and eating habits can affect the life quality and eating habits of their dogs, especially if owner has predisposed to obesity.	12
New public awareness for this huge problem and hopefully more informed audience;The social and scientific impact by putting in a scene the obesity problem among dogs and owners; The obesity in owners and dogs at the same time;	3
The use of social media for data creation was new to me. It has many advantages but also drawbacks and it is within this area in relation to constructions of web-based questionares where I have learnt the most.	1
(Blank)	2
**18) Did you experience any UNEXPECTED OUTCOMES of this obesity study initiative, the consortium collaboration or the publication process? If so, please describe** ***(quotes from respondents collated and counted by topic to ease interpretation)***	
Better experience in the publication process; Yes, if the study will be published in a high ranking journal (currently under peer-review for it) it will be an unexpected outcome.	2
I did not expect for owners to state that obesity is not a disease, and that many of them also stated that collaboration between human doctors and vets could not improve life quality; The low reported dog obesity rate in my country. Probably retalted to the above answer (the group of dog owner reached by the social media used); Lack of association between obesity in dog-owners and their dogs and large differences between countries;	3
I encountered difficulties in having a sufficient and significant number of answered questionnaires;	1
The differences in how we work academiccaly across countries;	1
No	8
(Blank)	7

(Supplementary materials 1 continued)	
**19) What are the most important likely societal / health IMPACTS of this obesity study initiative? Please describe in your own words** ***(quotes from respondents collated and counted by topic to ease interpretation)***	
Better studies being performed in the future on obesity at the human/animal interface - hopefully including more environmental and social aspects and stakeholder interactions during the whole project to assess uptake of the knowledge; This study is the first international study investigating obesity and possible interactions in dogs and their owners - differences and similarities have been elucidated and these are useful for future studies;	2
First of all, this study provides information about the situation in each different country and allows comparisons. The differences may be important to develop strategies adequated to each reality; More learning about the topic; Show facts/numbers of the problem in very simple way, to encourage changes of problematic people and animals, help to change; To pay attention of owners to the health not only they pets but themselfs as well;	4
Obesity can also affect dogs. The fact that a dog is obese could be because the owner has bad food habits or that he is also obese. One can influence the other; Obesity is increasingly important as a life threatening health problem. If the interactions of life style and eating behaviors between owner and dogs could be arranged, this regulation may be give the opportunity to solve the obesity as a public problem; The comparison between countries; The fact that pets and owners really have the obesity problem; To improve my knowledge of obesity in dogs; To raise awareness about the serious problem of obesity and inspire new transdisciplinary studies;	5
The awareness for direct and indirect obesity consequences, both among animals and owners; The results should be presented to people who completed the survey to correct the nutritional habits and attitudes of participants; To raise the importance of the obesity as a health problem;	3
(Blank)	5
**20) Any other comments that might help describe and evaluate the obesity study initiative?** ***(quotes from respondents***	
Good initiative! It would be good to keep the collaboration and to perform deeper analysis about this issue, in each of the countries involve, having as basis these results; Wonderful initiative; The evaluation should be very positive;	3
It would have been easier to anwer the questions after a publication of the manuscript. So far we have not communicated any results to any stakeholders.	1
The study is unfortunately unlikely to have much impact because it will just be published in a journal paper. A lot more would be required to ensure uptake of the knowledge and understanding of the complexity of the issue.	1
This study can be the start of a new signalment in human population that their own actions can affect everything that surrounds them.	1
No comments' or (Blank)	15
